# Interface-inspired formulation and molecular-level perspectives on heat conduction and energy storage of nanofluids

**DOI:** 10.1038/s41598-019-44054-0

**Published:** 2019-05-20

**Authors:** I. Carrillo-Berdugo, D. Zorrilla, J. Sánchez-Márquez, T. Aguilar, J. J. Gallardo, R. Gómez-Villarejo, R. Alcántara, C. Fernández-Lorenzo, J. Navas

**Affiliations:** Department of Physical Chemistry, Faculty of Sciences, University of Cádiz, Cádiz, Spain

**Keywords:** Physical chemistry, Other nanotechnology

## Abstract

Aiming for the introduction of stability requirements in nanofluids processing, an interface-based three-step method is proposed in this work. It is theory-based design framework for nanofluids that aims for a minimum tension at the solid-liquid interface by adjusting the polar and dispersive components of the base fluid to meet those of disperse nanomaterial. The method was successfully tested in the preparation of aqueous nanofluids containing single-walled carbon nanotubes that resulted to be stable and to provide good thermal properties, i.e. thermal conductivity increases by 79.5% and isobaric specific heat by 8.6% for a 0.087 vol.% load of nanotubes at 70 °C. Besides, a system for these nanofluids was modelled. It was found to be thermodynamically consistent and computationally efficient, providing consistent response to changes in the state variable temperature in a classical Molecular Dynamics environment. From an analysis of the spatial components of the heat flux autocorrelation function, using the equilibrium approach, it was possible to elucidate that heat conduction through the host fluid is enhanced by phonon propagation along nanotubes longitudinal axes. From an analysis of the structural features described by radial distribution functions, it was concluded that additional heat storage arises from the hydrophobic effect.

## Introduction

Nanofluids^1^ are a new generation of heat transfer fluids (HTF) that stand out for their heat transport and storage capabilities, greater than those of trivial fluids^2^. Their applicability for a better performance of heat exchangers in renewable power generation^3^ or microelectronics cooling^4^, among other applications, has promoted basic and applied research on nanofluids over the last two decades. Even though a lot of progress has been made, proper stability and heat transfer and storage mechanisms still remain as priority challenges in this field.

Stability is an essential requirement for preserving heat carriers and the sustaining these thermal properties. Previous works covering the stability of nanofluids^5–8^ are mainly focused on the dispersion gain with different methods and conditions or the changes in thermal properties as aggregation phenomena happen, but do not deal with the stability issue *a priori*. We realise the need for the rationalisation of new strategies that integrate stability in the formulation of nanofluids and, for that, this work introduces an interface-based three-step method. The inception of this three-step method stemmed from the poor stability that the existing procedures offer on the preparation of nanofluids with carbon nanotubes as disperse solid and water as base fluid. The method itself lies in: (i) the preparation of the nanomaterial, (ii) the preparation of a host fluid and (iii) the dispersion of the nanomaterial into the host fluid. The second step is innovative and involves an adjustment of the surface tension polar and dispersive components of the base fluid to meet those of the nanomaterial, so that the total tension at the interface is minimised. Conventional one-step and two-step methods for preparation^9^ do not include criteria for regulating the addition of surfactants, which in excess may increase viscosity and thermal resistance^10^, thus limiting heat transfer. A significant amount of time can be saved by rationalising the formulation of nanofluids under this method, because it avoids trial and error attempts when choosing surfactant concentration.

Heat transfer performance of nanofluids has been widely studied. The available literature reveals that thermal properties of nanofluids are influenced by the volume fraction^11,12^, nature^13,14^, morphology^15,16^ and size^17,18^ of the disperse particles, but the actual understanding of them is still quite limited due to the lack of a non-continuum heat transfer theory^2,19^. There are many formulations for the prediction of thermal conductivity in mixtures and composites^20^ and almost none of them are reliable for a general description of nanofluids because of discrepancies with experimental results. For isobaric specific heat, which is less studied, volume-weighted average and two-phase thermal equilibrium models^21^ are assumed to be valid while ignoring the role solid-liquid interactions. We explored new concepts that could be responsible of the anomalous thermal properties enhancement. A molecular model of the previously mentioned nanofluids was designed for its simulation in a Molecular Dynamics (MD) environment. Several runs were performed in order to validate the response of the model and to gain a molecular-level insight of its dynamics and structure for further comprehension on heat transport and storage mechanisms of these nanofluids. By doing so, the integrated experimental-theoretical approach aims to enable a clearer understanding on the behaviour of nanofluids.

## Experimental

### Formulation of nanofluids

The first step, preparation of the nanomaterial, involves any physical or chemical route leading to the acquisition of nano-structured solids. For this case, we started from commercial SWCNT (Sigma-Aldrich^©^ [6,5] chirality being most abundant, 0.78 nm ave. diameter, 1000 nm ave. length) prepared by CoMoCAT^®^ chemical vapour deposition.

The second step, preparation of a host fluid (a concept that comprises the base liquid and its additives), exploits some theoretical considerations about the thermodynamics of colloids^22^, whose formation is favoured if tension at the solid-liquid interface, γ_SL_, is minimized. Fowkes^23^ and Owens-Wendt^24^ defined γ_SL_ as

1 $${{\rm{\gamma }}}_{{\rm{SL}}}={{\rm{\gamma }}}_{{\rm{S}}}^{{\rm{p}}}+{{\rm{\gamma }}}_{{\rm{S}}}^{{\rm{d}}}+{{\rm{\gamma }}}_{{\rm{L}}}^{{\rm{p}}}+{{\rm{\gamma }}}_{{\rm{L}}}^{{\rm{d}}}-2\sqrt{{{\rm{\gamma }}}_{{\rm{S}}}^{{\rm{p}}}{{\rm{\gamma }}}_{{\rm{L}}}^{{\rm{p}}}}-2\sqrt{{{\rm{\gamma }}}_{{\rm{S}}}^{{\rm{d}}}{{\rm{\gamma }}}_{{\rm{L}}}^{{\rm{d}}}}$$

where ${{\rm{\gamma }}}_{{\rm{S}}}^{{\rm{p}}}$, ${{\rm{\gamma }}}_{{\rm{S}}}^{{\rm{d}}}$, ${{\rm{\gamma }}}_{{\rm{L}}}^{{\rm{p}}}$ and ${{\rm{\gamma }}}_{{\rm{L}}}^{{\rm{d}}}$ are the polar and dispersive components of the solid and liquid surface tensions, respectively. Thus, in order to minimize γ_SL_, the magnitudes of ${{\rm{\gamma }}}_{{\rm{S}}}^{{\rm{p}}}$ and ${{\rm{\gamma }}}_{{\rm{S}}}^{{\rm{d}}}$ and also the magnitudes of ${{\rm{\gamma }}}_{{\rm{L}}}^{{\rm{p}}}$ and ${{\rm{\gamma }}}_{{\rm{L}}}^{{\rm{d}}}$ should be as close as possible. Therefore, the basic idea is to modify and to adjust with additives the components of the base liquid to meet those of the disperse solid because in such case both phases are expected to be compatible and so a stable colloid can be prepared. Given the case of carbon nanotubes (${{\rm{\gamma }}}_{{\rm{CNT}}}^{{\rm{p}}}$ = 26.9 mN·m^−1^ and ${{\rm{\gamma }}}_{{\rm{CNT}}}^{{\rm{d}}}$ = 18.4 mN·m^−1^)^25^ in water (${{\rm{\gamma }}}_{{{\rm{H}}}_{2}{\rm{O}}}^{{\rm{p}}}$ = 54.8 mN·m^−1^ and ${{\rm{\gamma }}}_{{{\rm{H}}}_{2}{\rm{O}}}^{{\rm{d}}}$ = 22.1 mN·m^−1^), the components of the host fluid need to be decreased. This approach should be valid for SWCNT, MWCNT and carbon nanofibers, since their wetting properties are quite similar^25–27^. We prepared a series of aqueous standards with different concentrations (1.0·10^−3^, 2.0·10^−3^, 3.0·10^−3^, 4.0·10^−3^ and 5.0·10^−3^ vol.%) of Triton X-100 (Panreac^©^, surfactant for automatical analysis) and determined their polar and dispersive components. Although Triton X-100 surfactant has been previously used for the preparation of similar nanofluids^28–30^, its use has not been rationalised from this perspective. Young’s equation provides a relation of them with the contact angle θ_RL_ of a drop of these standards onto a surface (R) of reference:

2 $${{\rm{\gamma }}}_{{\rm{RL}}}={{\rm{\gamma }}}_{{\rm{R}}}-{{\rm{\gamma }}}_{{\rm{L}}}\,{\cos {\rm{\theta }}}_{{{\rm{S}}}_{{\rm{R}}}{\rm{L}}}=({{\rm{\gamma }}}_{{\rm{R}}}^{{\rm{p}}}+{{\rm{\gamma }}}_{{\rm{R}}}^{{\rm{d}}})-({{\rm{\gamma }}}_{{\rm{L}}}^{{\rm{p}}}+{{\rm{\gamma }}}_{{\rm{L}}}^{{\rm{d}}}){\cos {\rm{\theta }}}_{{\rm{RL}}}$$

The equalization of Eqs 1 and 2 lead to

3 $$\sqrt{{{\rm{\gamma }}}_{{\rm{R}}}^{{\rm{p}}}{{\rm{\gamma }}}_{{\rm{L}}}^{{\rm{p}}}}+\sqrt{{{\rm{\gamma }}}_{{\rm{R}}}^{{\rm{d}}}{{\rm{\gamma }}}_{{\rm{L}}}^{{\rm{d}}}}=\frac{{{\rm{\gamma }}}_{{\rm{L}}}(1+{\cos {\rm{\theta }}}_{{\rm{RL}}})}{2}$$

**Figure 1:**
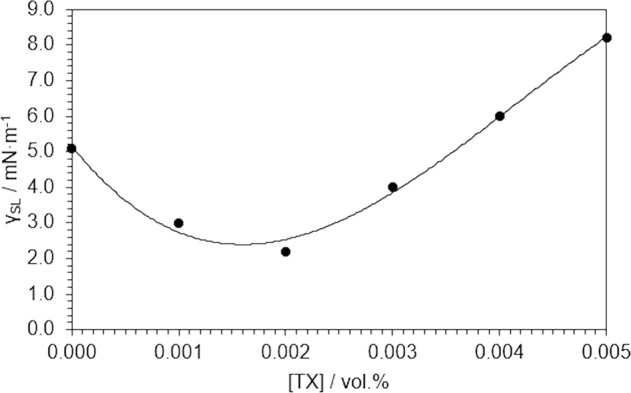
Dependence of the solid-liquid interface tension in the SWCNT/TX/H_2_O system for increasing volumetric fractions of Triton X-100.

A neat polished polytetrafluoroethylene (PTFE) stratum was chosen as surface of reference. Its polar and dispersive components were previously characterized by measuring the contact angles of a series of pure liquids whose polar and dispersive components are well known. At 25 °C and 1.0 atm in air atmosphere with 55% relative humidity, we found ${{\rm{\gamma }}}_{{\rm{R}}}^{{\rm{p}}}$ = 0.8 mN·m^−1^ y ${{\rm{\gamma }}}_{{\rm{R}}}^{{\rm{d}}}$ = 21.4 mN·m^−1^. Taking ${{\rm{\gamma }}}_{{\rm{R}}}^{{\rm{p}}}\simeq 0$ is a fair assumption that simplifies Eq. 3 into

4 $${{\rm{\gamma }}}_{{\rm{L}}}^{{\rm{d}}}=\frac{{({{\rm{\gamma }}}_{{\rm{L}}})}^{2}{(1+{\cos {\rm{\theta }}}_{{\rm{RL}}})}^{2}}{4{{\rm{\gamma }}}_{{\rm{R}}}^{{\rm{d}}}}$$

so that the dispersive components of each standard can be directly acquired from its absolute surface tension γ_L_, which is determined using the drop-weight method, and its contact angle θ_RL_, using a goniometer and an optical subsystem^31^. The same stratum and ambient conditions were used. Table 1 summarises the results for each standard. From these values, we predicted the dependence of the solid-liquid interface tension of the SWCNT/TX/H_2_O system for increasing volumetric fractions of Triton X-100. This is the plot in Fig. 1. From the fitting function, we determined a 1.7·10^−3^ vol.% of Triton X-100 in water that satisfies the minimum tension, which defines the host fluid of use.

**Table 1:** Absolute surface tension, contact angles and polar and dispersive component for a series of aqueous standards with different concentrations of Triton X-100.

[TX]/10^−3^ vol.%	γ_L_/mN·m^−1^	θ_RL_/°	${{\boldsymbol{\gamma }}}_{{\bf{L}}}^{{\bf{P}}}$/mN·m^−1^	${{\boldsymbol{\gamma }}}_{{\bf{L}}}^{{\bf{d}}}$/mN·m^−1^
0.0	76.9 ± 1.8	115.8 ± 1.6	54.8 ± 2.9	22.1 ± 2.2
1.0	71.2 ± 2.8	108.2 ± 3.2	43.2 ± 5.6	28.0 ± 4.9
2.0	65.8 ± 2.5	101.6 ± 0.6	33.5 ± 3.6	32.3 ± 2.6
3.0	61.3 ± 1.9	93.5 ± 4.0	22.6 ± 6.5	38.7 ± 6.2
4.0	56.7 ± 2.8	87.4 ± 0.7	15.6 ± 5.0	41.1 ± 4.2
5.0	53.1 ± 1.6	82.6 ± 0.9	11.1 ± 3.1	42.0 ± 2.6

The last step, dispersion of the nanomaterial into the host fluid, comprises the immersion, disaggregation and mixing processes. Approximately 1 mg of as-received powder-like SWCNT was dispersed within 100 ml of host fluid by means of stirring and pulsed sonication (Sonics&Materials^©^, VCX-500, 13 mm probe tip) set at 50% amplitude with alternating on/off bursts of 2 s length (47 W per pulse). Aliquots were extracted after four, six and eight hours of sonication and centrifuged at 7500 rpm for one hour in order to remove aggregates of large size. Supernatants were collected for characterisation and labelled as NF4, NF6 and NF8 (a nomenclature that references sonication time in hours). They were the nanofluids analysed in this work.

### Characterisation of nanofluids

Goodness criteria for nanofluid stability are constant load and size of heat carriers in suspension within the host fluid over a long-time period. The load was assessed in terms of spectral extinction using a UV-Vis extinction spectrometer (OceanOptics^©^, DH-2000-BAL light source and USB2000 + general purpose spectrometer), whereas the size was estimated by means of the dynamic light scattering technique (Malvern Instruments^©^, Zetasizer Nano ZS), both measured in triplicate every day during the first week and once a week during a full month. Also, the degree of dispersion was qualitatively studied with scanning electron microscopy (FEI^©^, Nova NanoSEM-450) images of SWCNT solid samples directly extracted from the host fluid by evaporation at 150 °C.

Since forced convection is the mechanism that rules macroscopic heat flow in a heat exchanger, quantifying the convection coefficient (h) is a minimum requirement to assess whether thermal performance is enhanced or not. The dimensionless Figure of Merit (FoM) h_nf_/h_bf_ may be used as an indicator to quantify the relative enhancement of thermal performance which is solely due to changes in those properties of the nanofluid (nf) with respect to its base fluid (bf), which is the HTF in use. According to the Dittus-Boelter equation^32^ (valid for fully developed turbulent flow in circular tubes under heating conditions), h_nf_/h_bf_ can be defined as

5 $$\frac{{{\rm{h}}}_{{\rm{nf}}}}{{{\rm{h}}}_{{\rm{bf}}}}={(\frac{{{\rm{\rho }}}_{{\rm{nf}}}}{{{\rm{\rho }}}_{{\rm{bf}}}})}^{0.8}{(\frac{{{\rm{\eta }}}_{{\rm{nf}}}}{{{\rm{\eta }}}_{{\rm{bf}}}})}^{-0.4}{(\frac{{{\rm{\kappa }}}_{{\rm{nf}}}}{{{\rm{\kappa }}}_{{\rm{bf}}}})}^{0.6}{(\frac{{{\rm{C}}}_{{{\rm{P}}}_{{\rm{nf}}}}}{{{\rm{C}}}_{{{\rm{P}}}_{{\rm{bf}}}}})}^{0.4}$$

where ρ is the density,η is the dynamic viscosity, κ is the thermal conductivity and C_p_ is the isobaric specific heat. Henceforth, these four properties were determined for each nanofluid. Density was measured using a pycnometer (Pobel^©^, 10 ml) at 25 °C and 1 atm. Dynamic viscosity was measured using a sine-wave viscometer (A&D Company^©^, SV-10) at 25 °C and 1 atm. Density and viscosity measurements were performed in triplicate. Thermal conductivity was measured with a transient hot-bridge (Linseis^©^, THB-100) operating at 25, 50 and 70 °C and 1 atm, with increasing input powers (10, 20 and 30 mW, respectively) for a better signal-to-noise ratio. Up to ten replicas were recorded for each sample and temperature. Lastly, isobaric specific heat was measured by means of temperature modulated differential scanning calorimetry (Netzsch^©^, DSC 214 Polyma), setting a temperature program that involves (i) a 5 °C/min ramp up to 40 °C, (ii) isothermal equilibration for 10 min, (iii) a 1 °C/min ramp down to 10 °C and (iv) a 1 °C/min ramp from 10 °C to 70 °C under temperature-modulated conditions with oscillations of ±1 °C amplitude and 1 min period.

## Computational Framework

### Modellisation of nanofluids

LAMMPS^33^ (Sandia National Labs, 31Mar17 version) was the code chosen to perform MD simulations for a comprehensive theoretical study of nanofluids. We configured models for the base fluid and the nanofluid using the molecular building tool Moltemplate^34^ (Jensen Lab, 23Oct17 version). Three templates (namely the TIP3P water molecule, the [6,5]-SWCNT unit cell and the Triton X-100 macromolecule in its all-anti conformation) were considered, with all their sites being explicit and a maximum occupancy of one atom per site. The nanofluid initial configuration was created by removing a cubic domain from an arrangement of equally spaced water molecules and then inserting a nanotube and two surfactant units. This ensures no overlap of atomic positions in the initial configuration and so that the simulation runs without instabilities. The OPLS-AA force field^35,36^ was used to compute bonding and non-bonding atomic interactions within the model in liquid phase simulations. It is described by the following functional of classical potentials

6 $$\begin{array}{rcl}{\rm{U}} & = & \sum _{{\rm{bonds}}}{{\rm{K}}}_{{\rm{l}}}{({\rm{l}}-{{\rm{l}}}_{0})}^{2}+\sum _{{\rm{angles}}}{{\rm{K}}}_{{\rm{\theta }}}{({\rm{\theta }}-{{\rm{\theta }}}_{0})}^{2}+\sum _{{\rm{dihedra}}}\sum _{{\rm{m}}}\frac{{{\rm{V}}}_{{\rm{m}}}}{2}{[1+\cos ({\rm{m}}{\rm{\varphi }})]}^{2}\\  &  & +\,\sum _{{\rm{ij}}}4{\epsilon }_{{\rm{ij}}}[{(\frac{{{\rm{\sigma }}}_{{\rm{ij}}}}{{{\rm{r}}}_{{\rm{ij}}}})}^{12}-{(\frac{{{\rm{\sigma }}}_{{\rm{ij}}}}{{{\rm{r}}}_{{\rm{ij}}}})}^{6}]+\sum _{{\rm{ij}}}\frac{{{\rm{q}}}_{{\rm{i}}}{{\rm{q}}}_{{\rm{j}}}}{4{{\rm{\pi }}{\rm{\varepsilon }}}_{0}{{\rm{r}}}_{{\rm{ij}}}}\end{array}$$

where K_1_ is the harmonic force constant of each two-body interactions of covalently bonded atoms stretching from their equilibrium length 1_0_; K_θ_ is the harmonic force constant of each three-body interactions of covalently bonded atoms bending from their equilibrium angle θ_0_; V_m_ are the torsion barriers for the dihedral angle ϕ of each four-body interactions of covalently bonded atoms rotating around a bond; r_ij_ is the distance between each pair non-bonded atoms, i and j, that interact as described by the energetics of the standard 12/6 Lennard-Jones potential, which is a function of the well depth *ε*_ij_ and the collision diameter σ_ij_, and the Coulomb potential, which is a function of the atomic charges q_i_ and q_j_. The parameters used for the OPLS-AA force field were adopted from literature^37–41^, as they were found to be compatible and transferable to our model. Lorentz-Berthelot combining rules were used to define the Lennard-Jones potential parameters for pair interactions between odd atoms, except for water-nanotube interactions, whose parameters were derived by Kaukonen *et al*.^38^ from van der Waals density functional (vdW-DF) calculations with no empirical parameters.

### Simulation of nanofluids

Equilibrium MD simulations were performed in a cubic environment with periodic boundary conditions imposed in all Cartesian directions. Velocities were rescaled with the Nosé-Hoover thermostat and barostat^42–44^ (with damps of 10 and 100 fs, respectively) and updated using the Verlet integration algorithm^45^ with a timestep of 1.0 fs. For the sake of computational time, Lennard-Jones pairwise interactions were limited to a cut-off distance of 10.0 Å, Ewald summation^46^ was applied to compute long-range electrostatics beyond that cut-off and TIP3P water geometry was constrained using the SHAKE algorithm, so that dynamics run freely but bonds and angles are reset to their equilibrium length and angular values after each timestep^47^.

Systems were initially forced to change their volume until density was equal to 1000 kg·m^−3^ (by doing so, velocities were rescaled for the atoms to match the box change and keep their natural motion), and then thermalized using the NVT ensemble at 25, 50 or 70 °C during 100 ps. Keeping up those conditions for 500 ps, transport coefficients were computed using the Green-Kubo formalisms^48,49^, considering a 10 ps correlation length and a 100 ps^−1^ sampling frequency. Autocorrelation functions were integrated using the trapezoidal rule numerical integration scheme. Lastly, the systems were pressurized using the isobaric-isothermal (NPT) ensemble at 1 atm during 100 ps. Now radial distribution functions (RDF) were computed, using the same correlation length and sampling frequency stated above. Simultaneously, density and isobaric specific heat values were computed every 10 ps up to 500 ps. Also, we dumped *.xyz files every 10 ps for the creation of 3D graphics in VMD^50^ (NIH Center for Macromolecular Modelling & Bioinformatics, 1.9.3 version).

## Results and Discussion

### Stability assessment

**Figure 2:**
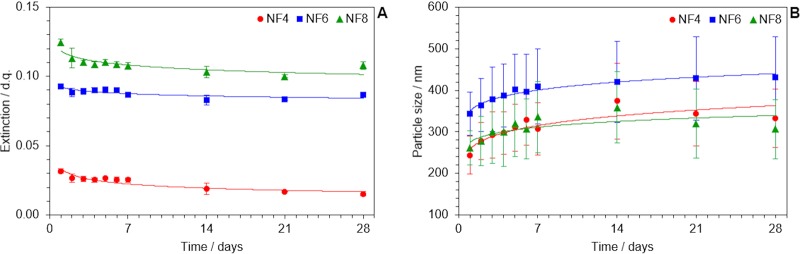
Evolution of (**A**) spectral extinction values at 566 nm and (**B**) average size and distribution width, in time, as indicators for stability.

Changes in load and size of heat carriers were monitored as stability indicators. The load of SWCNT in nanofluids was related to their spectral extinction, since the intensity of light that is extinguished due to absorption and scattering phenomena as radiation passes through the liquid sample and interacts with them. Figure 2A shows the evolution of extinction values registered at 566 nm during a full month period after nanofluids were prepared. Wavelength choice is not arbitrary but meaningfully justified by S_22_ transitions between van Hove singularities in [6,5]-SWCNT^51,52^, which is the most abundant chirality according to the information in supplier data sheet.

Attending to the initial values of extinction, the load of SWCNT introduced in the host fluid is greater for NF8, which is the sample treated under sonication for a larger time. However, there are no significant changes in the way they evolve. All nanofluids undergo a minimal aggregation process that is perceptible in a very early extinction decay that may be interpreted as a spontaneous change towards the equilibrium. Surface tension at the solid-liquid interface is low but still positive (see Fig. 1), so it can be expected from the system to reduce the interfacial surface. Indeed, we know from dynamic light scattering determinations, shown in Fig. 2B, that the average size (in terms of the hydrodynamic diameter) of heat carriers peaks a maximum value over the first two weeks in all cases. Size distribution width slightly increases over time, from about ±50 nm to ±100 nm. No phase segregation was observed by the end of the tests. So, the discussion above allows to conclude that NF4, NF6 and NF8 samples are stable once the equilibrium situation is reached, so their rheological and thermal properties are expected to be sustainable in time. What is more, the fulfilment of both stability criteria validates the theory-based design framework for nanofluids proposed in this three-step method for the preparation of stable carbon-based nanofluids in a polar host liquid.

**Figure 3:**
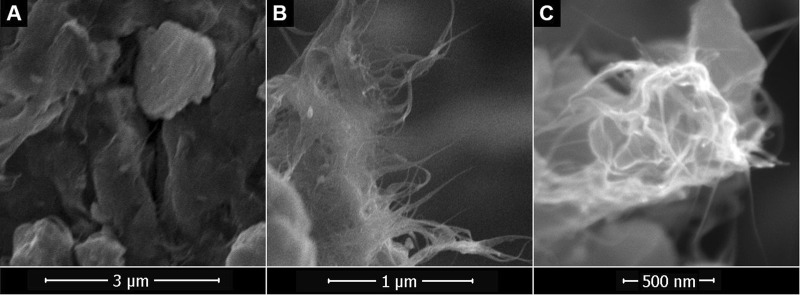
SEM images of SWCNT (**A**) from commercial powder and (**B**,**C**) from nanofluids.

Figure 3A contains SEM images of as-received SWCNT commercial powder, revealing that carbon nanotubes are originally entangled and compact. Alternatively, an array of randomly oriented carbon nanotubes can be seen in Fig. 3B, which corresponds to a powder-like sample extracted from the nanofluid. The entanglement is significantly reduced after sonication, even though they are forming some bundles after the extraction process. More sharpness and higher magnification in Fig. 3C allow the identification of small groups of carbon nanotubes in solid state samples. By comparing all SEM images, it can be clearly appreciated that the sonication treatment included in the preparation protocol provides a good degree of dispersion and that the properly controlled addition of stabilizers favours the availability of individual SWCNT or bundles with a small number of them.

### Thermal performance assessment

Density values for each nanofluid are available in Table 2. Given a standard volumetric flow in a heat exchanger system, that HTF with a higher density will provide a greater mass transfer coefficient and so a better heat transfer rate. This is numerically considered in the Dittus-Boelter equation introducing density values to the power of a positive exponent. However, we found density to increase up to 0.1% with respect to the base fluid, so it is no expected to be decisive for heat transfer enhancement. We estimated the volume fractions of solid (for discussions attending to the content of nanomaterial in each sample) from density by assuming that

7 $${\rm{\phi }}=\frac{{{\rm{\rho }}}_{{\rm{nf}}}-{{\rm{\rho }}}_{{\rm{bf}}}}{{{\rm{\rho }}}_{{\rm{nt}}}-{{\rm{\rho }}}_{{\rm{bf}}}}$$

where ρ_nf_, ρ_bf_ and ρ_nt_ are the densities of the nanofluid, the base fluid and the nanotube.

**Table 2:** Density and dynamic viscosity values of the base fluid and the nanofluids at 25 °C, together with the estimated volume fractions of solid (ρ_nt_ = 1800 ± 100 kg·m^−3^).

Sample ID	ρ/kg·m^−3^	φ/10^−2^ vol.%	η/mPa·s
Base fluid	996.4		0.89
Host fluid	996.4 ± 0.1	0.0	0.90 ± 0.01
NF4	996.6 ± 0.1	2.5 ± 1.3	0.93 ± 0.01
NF6	996.9 ± 0.1	6.2 ± 1.5	0.98 ± 0.01
NF8	997.1 ± 0.1	8.7 ± 1.7	1.01 ± 0.01

**Figure 4:**
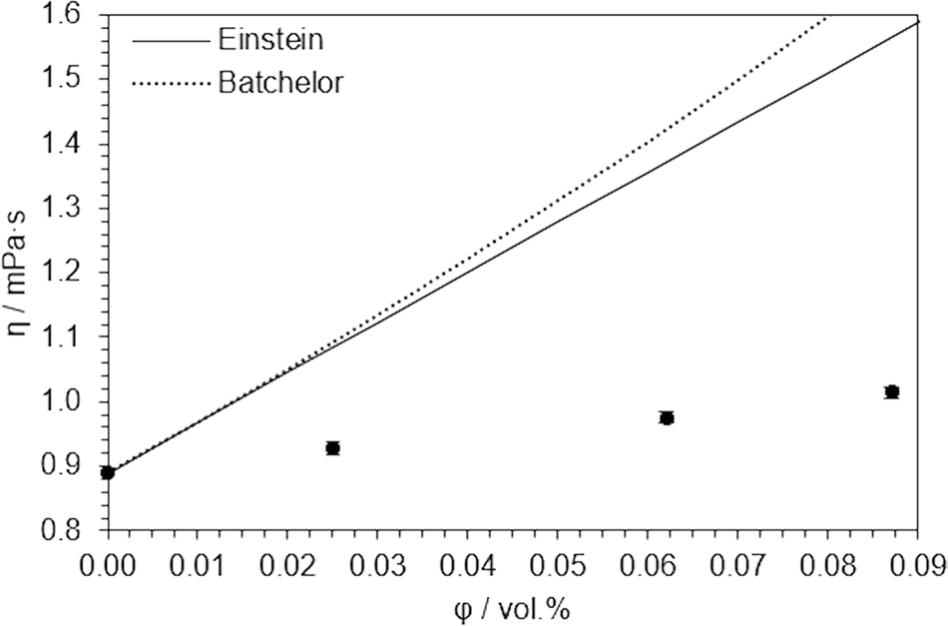
Dynamic viscosity values at 25 °C, as a function of SWCNT volume fraction, compared to those predicted by Einstein’s and Batchelor’s models.

Dynamic viscosity values are also represented in Table 2. It can be appreciated that they increase for increasing volume fractions of nanomaterial, up to 13.9%, which is still affordable for conventional pumping systems. We checked that the diluted amount of Triton X-100 does not cause a measurable difference in viscosity, so it is solely due to the presence of nanotubes. In Fig. 4 we compared our results with those predicted by those numerical models presented in Table 3. Einstein’s model^53^ for the viscosity of suspensions (Eq. 8) is based on the resolution of the hydrodynamic equations for suspensions of non-interacting rigid particles. Alternatively, Batchelor’s model^54^ (Eq. 9) takes into account the influence of two-particle interactions by introducing the Huggins coefficient k_H_, whose value was calculated by Berry et Russel^55^ as 2/5 for interactions between rigid rods under steady shear flow conditions. In both cases the intrinsic viscosity {η} parameter is included, which is sensitive to the particle shape. Its theoretical expression, which can be found in literature^56^, is a function of the particle diameter-to-length ratio d_np_/L_np_. For the calculation of such a ratio, average diameter and length of SWCNT (available in the supplier data sheet) were considered. Despite this, none of these models was found to fit our experimental data, and the main limitation here may be the fact of nanotubes being considered as rigid bodies (as both models are constructed on this assumption) with a diameter-to-length ratio of 7.8·10^−4^ whereas they can be stacked or partially folded in suspension, which should be properly represented by a larger aspect ratio. Still, the main conclusion that can be drawn from the comparison of these models in Fig. 4 is that the rheological behaviour of these nanofluids is ‘better’ described (since it is reasonably linear) by the Einstein’s model and so that SWCNT, for low volume fractions in a host fluid with optimum surface tension, are more likely to behave as non-interacting (but not necessarily rigid) particles. This means minimum aggregation and maximum availability of heat carriers in suspension.

**Table 3:** Models to calculate the effective viscosity (Eqs 8 and 9) and thermal conductivity (Eqs 10 and 11), as a function of the volume fraction and shape of the disperse solid.

Reference	Equation
Einstein^53^	${{\rm{\eta }}}_{{\rm{eff}}}={{\rm{\eta }}}_{{\rm{bf}}}(1+\{{\rm{\eta }}\}{\rm{\phi }})$(9)
Batchelor^54^	${{\rm{\eta }}}_{{\rm{eff}}}={{\rm{\eta }}}_{{\rm{bf}}}(1+\{{\rm{\eta }}\}{\rm{\phi }}+{{\rm{k}}}_{{\rm{H}}}{\{{\rm{\eta }}\}}^{2}{{\rm{\phi }}}^{2})$ (10)
Hamilton-Crosser^57^	${{\rm{\kappa }}}_{{\rm{eff}}}={{\rm{\kappa }}}_{{\rm{bf}}}\frac{{{\rm{\kappa }}}_{{\rm{np}}}+(3{{\rm{\Psi }}}^{-1}-1)({{\rm{\kappa }}}_{{\rm{bf}}}-{\rm{\phi }}({{\rm{\kappa }}}_{{\rm{bf}}}-{{\rm{\kappa }}}_{{\rm{np}}}))}{{{\rm{\kappa }}}_{{\rm{np}}}+(3{{\rm{\Psi }}}^{-1}-1){{\rm{\kappa }}}_{{\rm{bf}}}+{\rm{\phi }}({{\rm{\kappa }}}_{{\rm{bf}}}-{{\rm{\kappa }}}_{{\rm{np}}})}$ (11)
Yamada-Ota^58^	${{\rm{\kappa }}}_{{\rm{eff}}}={{\rm{\kappa }}}_{{\rm{bf}}}\frac{{{\rm{\kappa }}}_{{\rm{np}}}{/{\rm{\kappa }}}_{{\rm{bf}}}+2{{\rm{\phi }}}^{0.2}{{\rm{L}}}_{{\rm{np}}}{/{\rm{d}}}_{{\rm{np}}}(1-{\rm{\phi }}(1-{{\rm{\kappa }}}_{{\rm{np}}}{/{\rm{\kappa }}}_{{\rm{bf}}}))}{{{\rm{\kappa }}}_{{\rm{np}}}{/{\rm{\kappa }}}_{{\rm{bf}}}+2{{\rm{\phi }}}^{0.2}{{\rm{L}}}_{{\rm{np}}}{/{\rm{d}}}_{{\rm{np}}}+{\rm{\phi }}(1-{{\rm{\kappa }}}_{{\rm{np}}}{/{\rm{\kappa }}}_{{\rm{bf}}})}$ (12)

**Figure 5:**
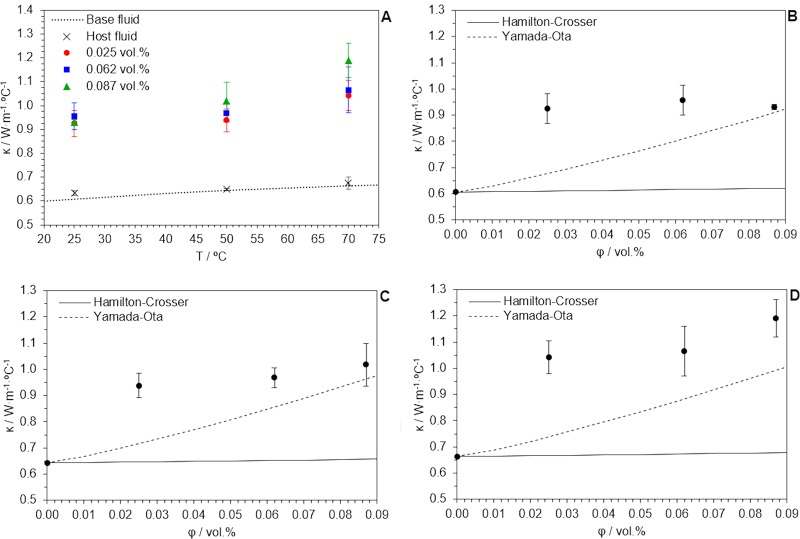
(**A**) Measured thermal conductivity values, as a function of temperature and volume fraction of disperse solid, compared to those predicted by Hamilton-Crosser’s, and Yamada-Ota’s models, at (**B**) 25 °C, (**C**) 50 °C and (**D**) 70 °C.

Figure 5A shows thermal conductivity values of the base fluid, the host fluid and SWCNT/TX/H_2_O nanofluids, as a function of temperature. As it increases, that nanofluid with the highest volume fraction of SWCNT seems to be more sensitive to changes in temperature, peaking a 79.5% enhancement in thermal conductivity at 70 °C. These enhancements are better than expected by numerical predictions using models from Table 3, at any temperature. This is better appreciated in Fig. 5B–D. Hamilton-Crosser’s model^57^ (Eq. 11) is supposed to calculate the effective thermal conductivity of a biphasic mixture as a function of their individual thermal conductivities by considering the sphericity Ψ and the volume fraction φ of the disperse particles but, for this case, it estimates an enhancement below 2.0%. The applicability of this model is hardly limited by its poor approach to non-spheroid particle shapes. Aiming for a wider applicability of thermal conductivity predicting formulae, Yamada-Ota’s model^58^ (Eq. 12) was originally proposed for parallelepiped particle suspensions by introducing the length-to-diameter ratio L_np_/d_np_. For the calculation of such a ratio, once again, average length and diameter of SWCNT (available in the supplier data sheet) were considered. The prediction is closer indeed, but this model also fails to predict thermal conductivities of the nanofluids presented in this study. A discussion of transport properties of nanofluids on the basis of numerical models is clearly limited because they are systems of high complexity that cannot be properly understood as common solid-liquid mixtures. For that we think the use of MD simulations helps for a better understanding of these systems and their properties.

Now considering the low volume fractions involved in this work, these are, to our knowledge, the highest enhancements ever reported in literature for nanofluids containing carbon nanotubes. For instance, Choi *et al*.^59^ reported a 159% enhancement of thermal conductivity in a synthetic poly-α-olefin oil with a 1.0 vol.% load of MWCNT at room temperature. Ding *et al*.^60^ also studied the thermal behaviour of nanofluids with a 1.0 vol.% load of MWCNT in ethylene glycol as reported an 80% enhancement of thermal conductivity. Alternatively, Harish *et al*.^61^ reported a 14.8% enhancement for a 0.20 vol.% load of SWCNT in ethylene glycol at 25 °C, whereas Sabiha *et al*.^62^ reported a 36.4% enhancement for a 0.25 vol.% load of SWCNT in water at 60 °C. Our achievement, as far as we can conclude, is a consequence of the availability of properly dispersed and stabilized heat carriers.

**Figure 6:**
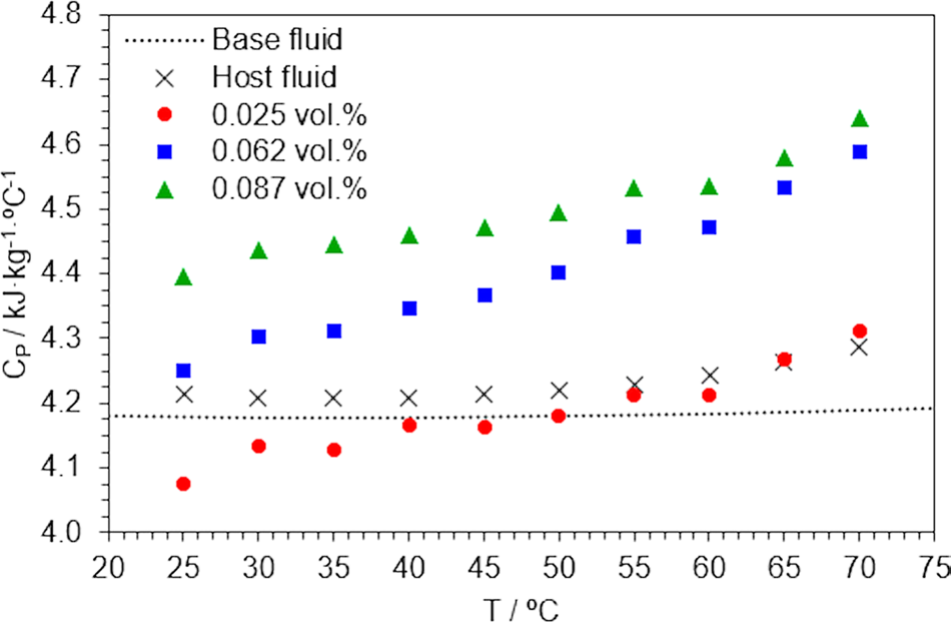
Isobaric specific heat values, as a function of temperature and volume fraction of disperse solid.

The values of isobaric specific heat are discussed. This property plays an important role in heat transport phenomena, as it determines the thermal energy storage capacity of the HTF in use. The specific heat of nanofluids is expected to decrease for increasing volume fractions, as the specific heat of solids is generally lower than that of liquids, but some experimental observations are opposed to this assumption^63^. Figure 6 illustrates that the isobaric specific heat of NF4 sample is lower than those of the base fluid and the host fluid at low temperatures, but it significantly increases with temperature. NF6 and NF8 samples have higher specific heats over the entire range. A maximum 8.6% enhancement was found for the 0.087 vol.% nanofluids at 70 °C.

**Figure 7:**
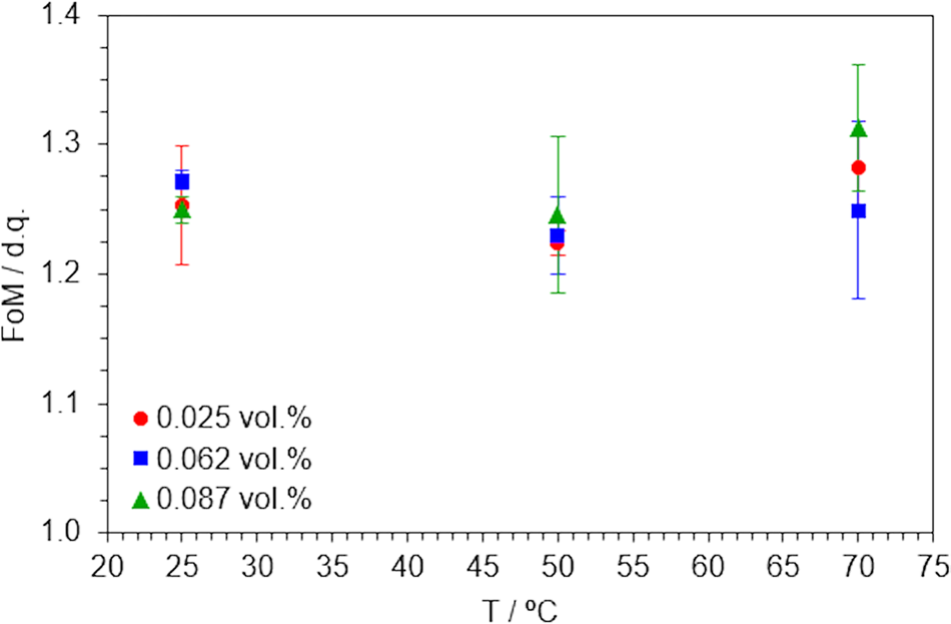
Expected enhancements in convective heat transfer, in terms of the FoM, as a function of temperature and volume fraction of disperse solid.

Contributions to heat transfer from these parameters must be assessed attending to a single criterion, what gives sense to the FoM defined in Eq. 5. Figure 7 gathers the values calculated for each temperature and volume fraction according to this formula. Any FoM value higher than 1.0 implies that nanofluids enhances the heat transfer process with respect to the base fluid. Best performance was found for the 0.087 vol.% SWCNT/TX/H_2_O nanofluid, which is more efficient than water by 31.3% under heating conditions at 70 °C, but still the criterion is satisfied in all cases, so any of these nanofluids could be a choice of preference over its base fluid to be used as HTF for thermal management. In overall terms, the conception of the three-step method leads us to the production of a whole series of SWCNT/TX/H_2_O nanofluids that were found to be stable and to provide an exceptional heat transfer efficiency for both conduction and convection processes according to the findings of an exhaustive systematic characterisation. So, the three-step method proposed here can be a promising advance in the field of nanofluids.

### Understanding heat conduction

**Figure 8:**
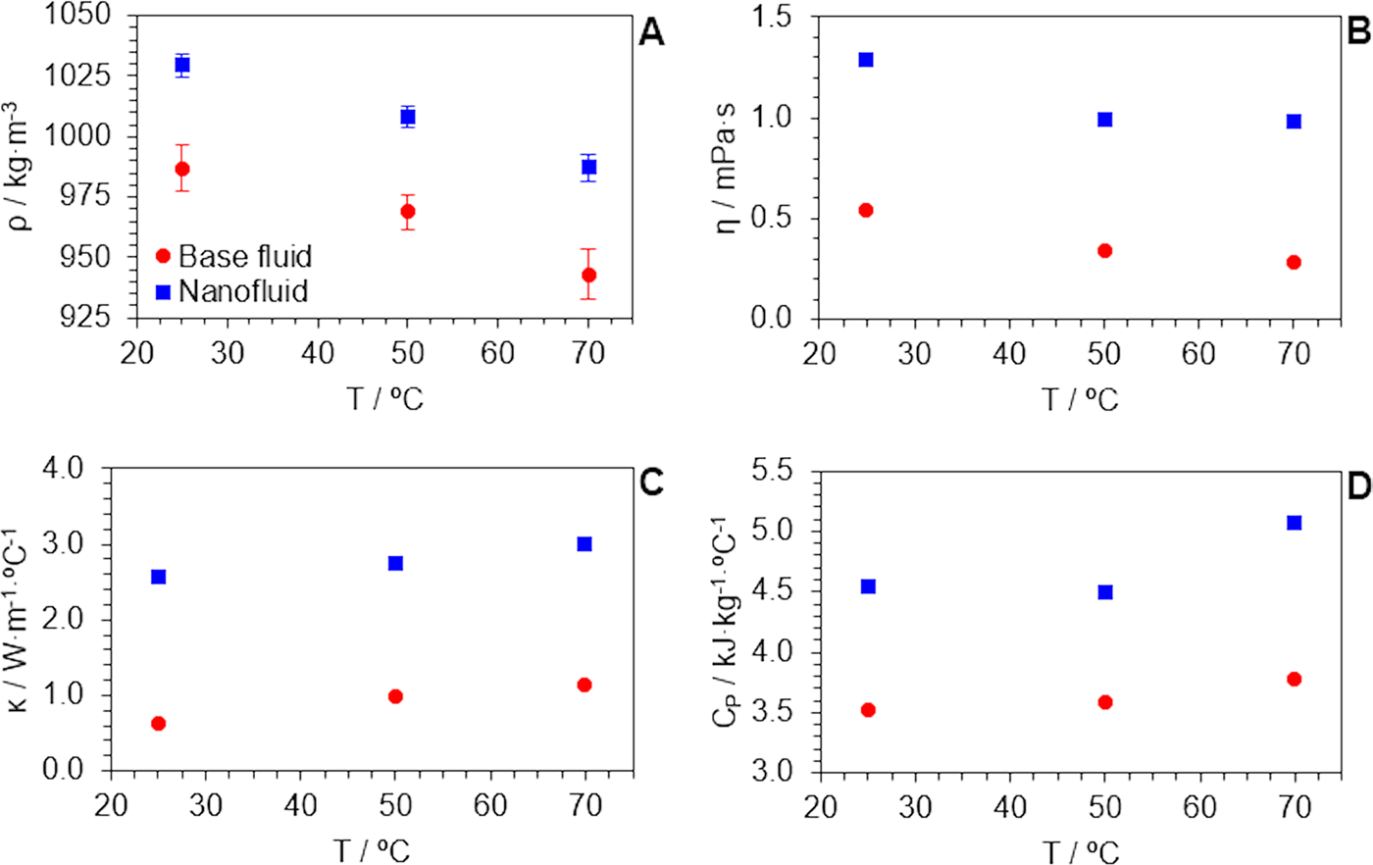
Computed values for (**A**) density, (**B**) dynamic viscosity, (**C**) thermal conductivity and (**D**) isobaric specific heat, for both the base fluid and the nanofluid models, as a function of temperature.

Before using the simulation approach for understanding and rationalising a system, it is important to validate the model with well-known experimental results. Figure 8A–D show computed values of the physical properties for both the base fluid and the nanofluid models, and how the presence of a carbon nanotube induces changes in the properties of the base fluid, which are clearly exalted. These values are slightly overestimated if compared with empirical evidences, and this is due to the limitation of the model size (the occupancy of carbon nanotube in the simulation box is equivalent to 1.4 vol.%, which is higher than experimental volume fractions by two orders of magnitude). Note that it is built as a finite box under periodic boundary conditions that pretends to emulate an infinite bulk domain. The major concern here was the number of atoms within that finite box and for that a trade-off between system representativeness (i.e. the nanotube must be a minor component with respect to whole number of water molecules), calculation convergence and processing time was required for the simulation to be feasible. However, it is noteworthy that thermal conductivity (see Figs 5A and 8C) and isobaric specific heat (see Figs 6 and 8D) values follow experimental-like trends. This proves that the model offers good response to state variable changes and good representativeness of nanofluids, and also demonstrates the thermodynamic consistency of the chosen force field parameters for the simulation of its dynamics^64^. All these facts lead to a successful and unambiguous validation of the model for further analysis.

Defining the mechanisms that rule heat transport phenomena in nanofluids is a key factor towards the comprehension of their thermal properties. This is a controversial issue among researchers. Keblinski *et al*.^65^, on the basis of MD simulations using a hypothetical nanofluid, suggested that the major contribution to thermal conductivity is the ballistic heat transport through in solid nanoparticles promoted by phonons and also that solid-liquid interfaces may influence the overall process in nanofluids. Evans *et al*.^66^, following a similar scheme, stated that thermal conductivity enhancements are not associated to hydrodynamic effects induced by Brownian motion. Alternatively, Sarkar *et al*.^67^, using copper-argon system model, concluded that is not the Brownian motion of nanoparticles but the enhanced movement of the surrounding base fluid atoms what promotes localized heat transport. These proposals, although they are far from being conciliated in a heat transfer theory for nanofluids, share a common point: nanoparticles modify the natural dynamics of the base fluid.

Our thermal conductivity results encouraged the need for understanding the underlying heat conduction mechanisms in nanofluids. In this work, we proceeded with the equilibrium approach for the analysis of heat conduction through nanofluids using a model whose dynamics were observed in a MD environment. For such approach, the Green-Kubo formalism^48,49^ provides the following time-correlation function to calculate thermal conductivity

8 $${\rm{\kappa }}=\frac{1}{3{{\rm{VT}}}^{2}{{\rm{k}}}_{{\rm{B}}}}{\int }_{0}^{\infty }\langle {\rm{J}}({\rm{t}})\cdot {\rm{J}}(0)\rangle \,{\rm{dt}}$$

where V is the system volume, T is the system temperature, k_B_ is the Boltzmann constant and 〈J(t) · J(0)〉 is the heat flux autocorrelation function (HFAF). This function describes how long it takes for the fluctuations (about zero at equilibrium) of the heat flux vector J(t) to dissipate, which implicitly contains information about the heat conduction process through the nanofluid^68^.

**Figure 9:**
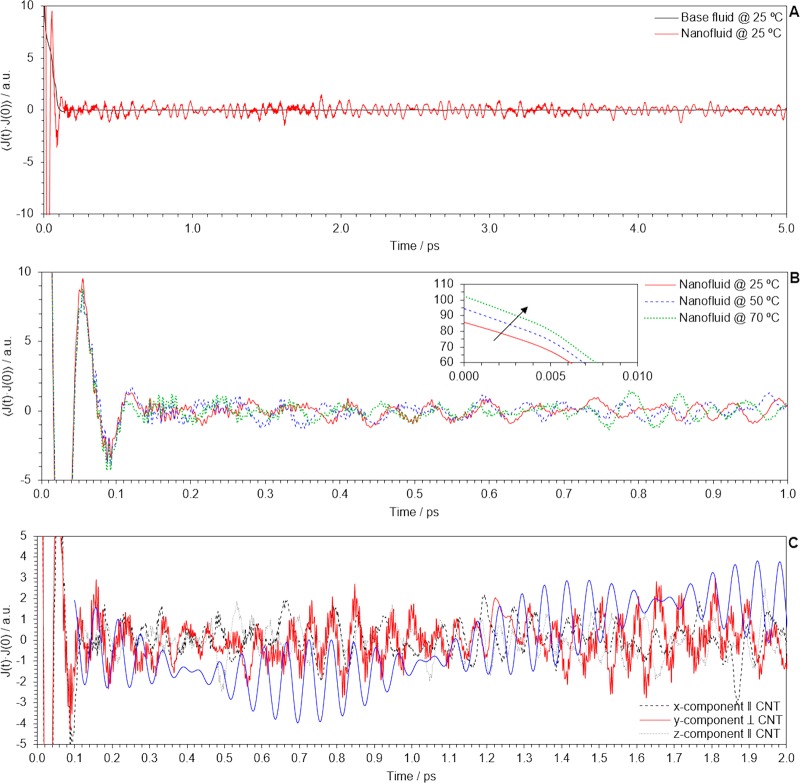
(**A**) HFAF for the base fluid and the nanofluid, at the same temperature; (**B**) HFAF of the nanofluid for increasing temperatures; (**C**) Components of the HFAF of the nanofluid.

Figure 9A contains the HFAF of the base fluid and the nanofluid at the same temperature. Decay pattern to zero was found to be monotone for the base fluid but oscillating for the nanofluid, in agreement with Keblinski *et al*. and Sarkar *et al*.^65,67^ Correlation lasted longer due to these thermal fluctuations, thus leading to a higher thermal conductivity of the nanofluid upon integration. For increasing temperatures, as it is shown in Fig. 9B, the decay ratio is lower and that explains why thermal conductivity of these nanofluids is sensitive to changes of this state variable. Figure 9C shows the spatial components of the HFAF (this is J_x_(t) · J_x_(0), J_y_(t) · J_y_(0) and J_z_(t) · J_z_(0)) for the nanofluid system at the highest temperature. Thermal fluctuations within the x- and z-directions, which are orthogonal to the nanotube longitudinal axis, depict a random sequence but those within the y-direction, which contains the nanotube longitudinal axis, depict some periodic features that resemble much of ‘heat beats’, as it is shown in Fig. 9C. We verified that the frequency of those features in the HFAF fits with that expected for the superposition of resonant n = 1 and n = 2 vibration modes of phonons in a well whose boundary width is equal to the nanotube length^69^, together with a higher frequency pattern that matches the oscillations in the HFAF of the base fluid, but with a much larger amplitude. This could be due to phonon-enhanced water flow, as we found water molecules occupy the nanotube inner cavity, as is discussed below. For better appreciation, we found convenient to plot the superposition of the temporal parts of the classical waves with the matching frequencies.

Given the statements above, we concluded that presence of nanotubes disturbs the natural dynamics of the base fluid by increasing the phonon mean free path along the nanotube longitudinal axis (local anisotropy), thus promoting the correlation of thermal fluctuations and leading to a higher thermal conductivity. If extended to a bulk system, we suggest that coherent and consecutive phonon propagation events between randomly oriented nanotubes (average isotropy), together with phonon-enhanced water flow, are responsible of the macroscopic thermal conductivity of the nanofluid. It is important to note that it is the Green-Kubo equilibrium methodology what provides access to this information (which is otherwise missed by non-equilibrium methodologies that impose a temperature gradient in a certain direction) and so we assert that it meets the requirement for the analysis of heat conduction at a non-continuum scale in nanofluids.

### Understanding heat storage

The enhancement of isobaric specific heat in nanofluids has received much less attention than thermal conductivity from theoretical research. Shin *et al*.^70^, in a consideration on the mechanisms responsible of this anomalous enhancement, proposed three independent modes for heat storage in nanofluids: (i) higher specific heat of nanoparticles compared to the bulk solid, due to the high surface energy per unit mass; (ii) additional storage arising from solid-liquid interactions at interfaces; (iii) layering of liquid molecules around nanoparticles, configuring a solid-like layer with higher specific heat than the bulk liquid. Jo *et al*.^71^, on the basis of MD simulations, were able to estimate the thickness and the density of a compressed liquid layer on the surface of graphite nanoparticles in a binary carbonate molten-salt. Navas *et al*.^13^, by analysing radial and spatial distribution functions from MD simulations, observed the arrangement of base fluid (a eutectic mixture of biphenyl and diphenyl oxide) molecules in a rigid layer surrounding copper and nickel nanoparticles and also concluded that the nature of interactions determines how tightly the layer is bound to the surface and how that boost heat transfer through the interface.

**Figure 10:**
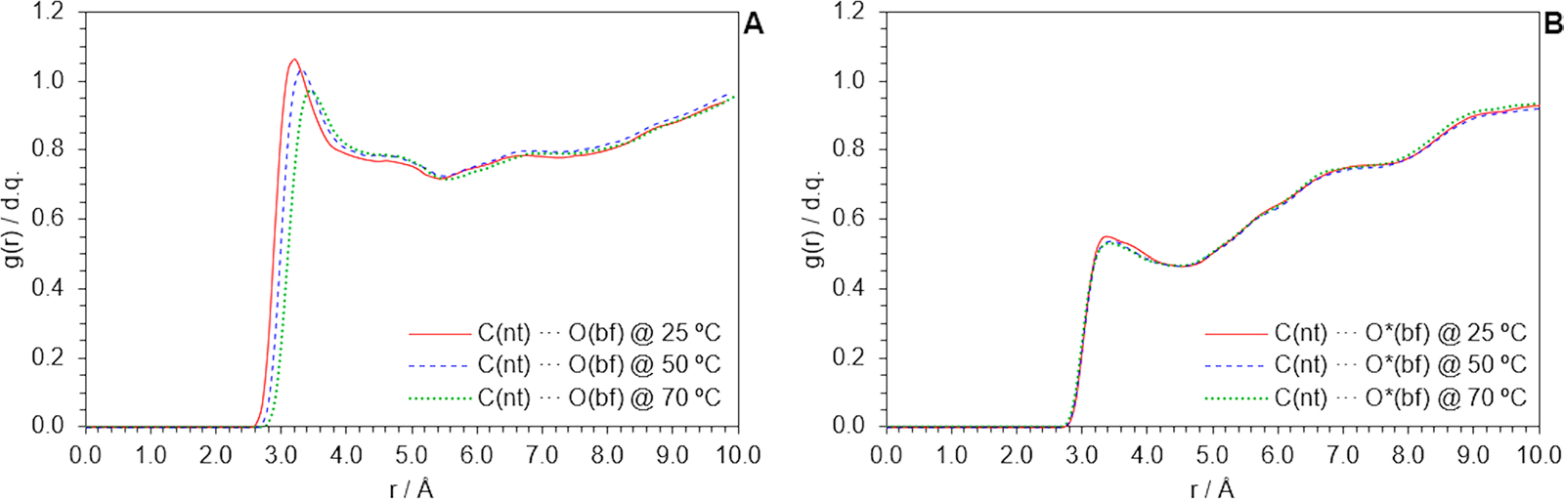
RDF for the C_nt_ … O_bf_ pair, acquired from the nanofluid model by MD simulations at different temperatures, including (**A**) or excluding (**B**) the presence of water inside the nanotube.

**Figure 11:**
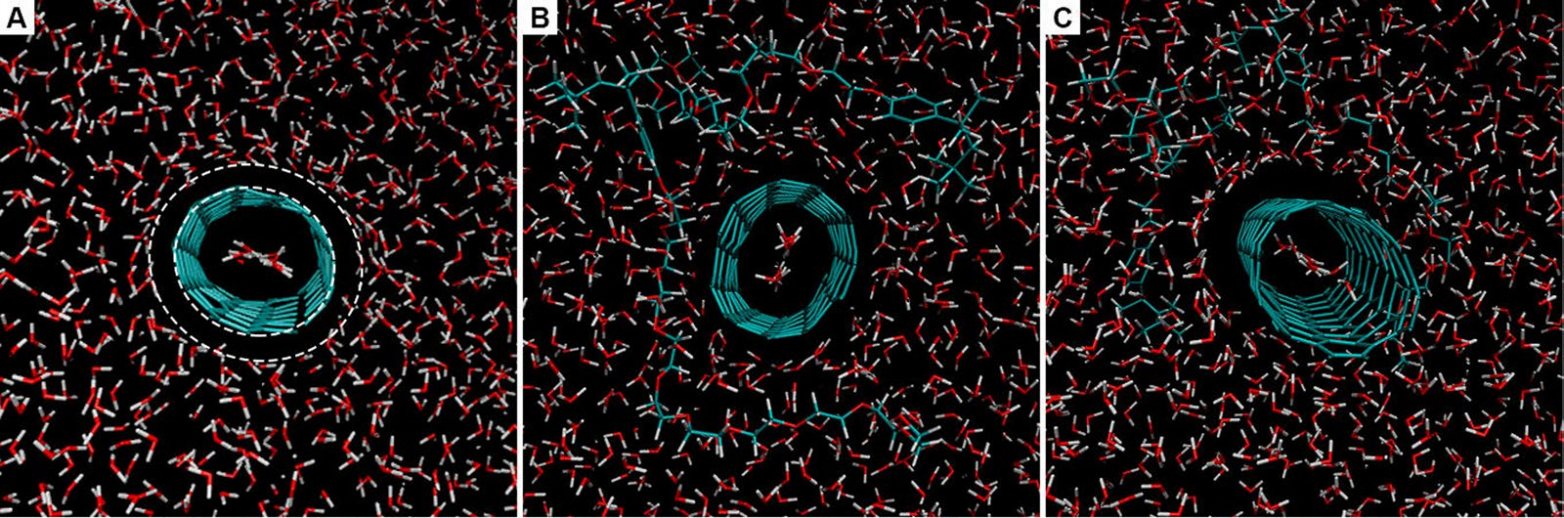
Cross sections of the nanofluid model for illustration of (**A**) water flow through the nanotube and the hydrophobic effect on water molecules around the nanotube, and (**B**,**C**) how the ‘solid-like’ layer of base fluid blocks the proximity of Triton X-100 surfactant.

Since our model was found to reproduce that anomalous heat storage enhancement, we proceeded with an analysis of the equilibrium molecular architecture from MD simulations. Computed RDFs for the C_nt_ ··· O_bf_ pair, which are shown in Fig. 10A, revealed an anomalous population of water molecules within a virtual shell defined between 3.0–4.0 Å from the nanotube surface. This accounts for both water inside and outside the nanotube (see Fig. 11A for better appreciation). C_nt_ ··· O_bf_ RDFs were again computed for a system that was forced not to have water molecules inside the nanotube and plotted in Fig. 10B. Number density of water molecules outside the nanotube is not consistent with sharp layering due to specific interactions but with some short-range order induced by the presence of the nanotube, that is distinguishable of bulk disorder and not altered by temperature (although loss of order would be expected as kinetic energy increases, it seems not to be significant for the studied range of temperatures). This observation allows a molecular interpretation of the isobaric specific heat increase in terms of the ‘hydrophobic effect’^72,73^: since nanotubes are non-polar, its inclusion in water forces the formation of a cage of molecules around them (i.e. some sort of clathrate), with those molecules being in a more ‘solid-like’ (yet dynamic) situation due to constrains arising from a higher number atom-atom interactions per molecule. Compared to liquid water, more heat is required to break up that structure, hence isobaric specific heat is increased.

On the other hand, RDF revealed no significant number density for any interaction of Triton X-100 atoms within a 10.0 Å cut-off radius from nanotube carbon atoms and, given its spatial configuration (see Fig. 11B,C), it seems not to be fully adsorbed onto the surface. The polyether chain is indeed preferentially located in the aqueous phase; as nanotube carbon atoms exhibit no natural charge separation, the extension of its adsorption is limited to van der Waals interactions to the nanotube surface, which are weaker than electrostatics interactions making water more competitive for solvation. The non-polar head is more akin to the surface but still attached to the polyether chain, which makes it more steric demanding and unable to approach towards the surface. The cumulative result of the above-stated forces suggests that Triton X-100 is partially adsorbed, yet located in the vicinity of the solid-liquid interface and altering its energetics. This fact may serve as a piece of evidence to conclude that the stabilisation provided to SWCNT/TX/H_2_O nanofluids is not arising from electrosteric events but from its influence on those energetics, changing cohesive forces and thus change surface tension.

## Conclusions

Next stage on the roadmap of nanofluids is the improvement of colloidal stability for sustainable thermal properties. An interface-based three-step method, arising from the rationalisation of the minimum tension at the interface between the disperse solid and the host fluid, was proposed in this paper. It was tested for the preparation of aqueous nanofluids containing single-walled carbon nanotubes, using the non-ionic surfactant Triton X-100 for the purpose of adjusting the polar and dispersive components of the base liquid until convergence with those of the disperse solid, thus making both phases compatible. Characterisation procedures revealed good dispersion and stability, in terms of constant particle load and size distribution, during at least one month. No significant changes in rheological properties were found but an extraordinary 79.5% enhancement in thermal conductivity and 8.6% in isobaric specific heat were observed for that nanofluid with a 0.087 vol.% load of nanotubes in suspension at 70 °C. Also, the heat convection process, as assessed by the Dittus-Boelter Figure of Merit, was enhanced by 31.3% for same nanofluid at the same temperature. Overall stability and thermal performance endorse the proposed three-step method and the method itself fulfils the target of sustainable thermal properties.

These results promoted the interest for the understanding of structural and dynamic features explaining thermal properties of nanofluids. For that, a homologous nanofluid system was modelled and validated for its simulation in a classical Molecular Dynamics environment. The spatial components of the heat flux autocorrelation function of the system revealed that heat conduction is significantly enhanced along the nanotube longitudinal axis due to a larger phonon mean free path. We suggested that consecutive phonon propagation events between randomly oriented nanotubes are responsible of the macroscopic thermal conductivity observed for these type of nanofluids. It was possible to elucidate from an analysis of the radial distribution functions that liquid layering phenomena occurs and may be related to isobaric specific heat enhancement due to the hydrophobic effect. Also, that Triton X-100 surfactant is partially adsorbed on the solid-liquid interface and so the stabilisation mechanism is not arising from electrosteric hindrance but from surface tension minimisation. Here modelling and simulation have supported experimental characterisation by providing information that cannot be obtained empirically.
